# Cloning of *Thalassiosira pseudonana’s* Mitochondrial Genome in *Saccharomyces cerevisiae* and *Escherichia coli*

**DOI:** 10.3390/biology9110358

**Published:** 2020-10-26

**Authors:** Ryan R. Cochrane, Stephanie L. Brumwell, Arina Shrestha, Daniel J. Giguere, Samir Hamadache, Gregory B. Gloor, David R. Edgell, Bogumil J. Karas

**Affiliations:** Department of Biochemistry, Schulich School of Medicine and Dentistry, The University of Western Ontario, London, ON N6A 5C1, Canada; rcochra3@uwo.ca (R.R.C.); sbrumwe2@uwo.ca (S.L.B.); ashrest7@uwo.ca (A.S.); dgiguer@uwo.ca (D.J.G.); shamadac@uwo.ca (S.H.); ggloor@uwo.ca (G.B.G.); dedgell@uwo.ca (D.R.E.)

**Keywords:** mitochondria, diatoms, *Thalassiosira pseudonana*, *Phaeodactylum tricornutum*, *Saccharomyces cerevisiae*, RNA sequencing, yeast-mediated cloning, organelle, organelle genome engineering, synthetic biology

## Abstract

**Simple Summary:**

One of the challenges in the emerging field of synthetic biology is engineering organelle genomes. Creating synthetic organelle genomes can open the door to a wide range of applications, such as improving crop yields, treating mitochondrial diseases, or manufacturing high-value chemicals in an environmentally sustainable way. Organelles are tiny biological machines that work inside of living cells. Mitochondria, for example, are responsible for harvesting sugar to create energy for the cell. In previous work, we demonstrated a method to make copies of an alga mitochondrial genome using yeast and bacteria. Algae are of industrial interest for their potential to produce and store large quantities of biofuels and nutritional ingredients. Here, we applied the same approach to copy the mitochondrial genome of a related alga. Although the cloning of this mitochondrial genome in yeast using the previously developed method was possible, the properties of this genome may make it more susceptible to mutations during propagation in bacteria. This work expands our understanding of potential hurdles that can be encountered when cloning and propagating synthetic organelle genomes in host organisms.

**Abstract:**

Algae are attractive organisms for biotechnology applications such as the production of biofuels, medicines, and other high-value compounds due to their genetic diversity, varied physical characteristics, and metabolic processes. As new species are being domesticated, rapid nuclear and organelle genome engineering methods need to be developed or optimized. To that end, we have previously demonstrated that the mitochondrial genome of microalgae *Phaeodactylum tricornutum* can be cloned and engineered in *Saccharomyces cerevisiae* and *Escherichia coli*. Here, we show that the same approach can be used to clone mitochondrial genomes of another microalga, *Thalassiosira pseudonana.* We have demonstrated that these genomes can be cloned in *S. cerevisiae* as easily as those of *P. tricornutum*, but they are less stable when propagated in *E. coli*. Specifically, after approximately 60 generations of propagation in *E. coli*, 17% of cloned *T. pseudonana* mitochondrial genomes contained deletions compared to 0% of previously cloned *P. tricornutum* mitochondrial genomes. This genome instability is potentially due to the lower G+C DNA content of *T. pseudonana* (30%) compared to *P. tricornutum* (35%). Consequently, the previously established method can be applied to clone *T. pseudonana*’s mitochondrial genome, however, more frequent analyses of genome integrity will be required following propagation in *E. coli* prior to use in downstream applications.

## 1. Introduction

Recent advancements in DNA sequencing and synthesis resulted in the development of a powerful set of biotechnology tools that can help to address global challenges in food and water sustainability, medicine production, and eco-friendly energies. Many potential organisms are under investigation for desirable properties useful for biotechnology applications. One attractive candidate is *Thalassiosira pseudonana*. This model centric diatom is naturally found in oceanic water and plays a significant role in global carbon cycling and combatting climate change [1,2]. In addition, its silica frustule encasement is suitable for nanotechnologies and drug delivery [3,4]. Due to the growing interest in *T. pseudonana*, its nuclear, mitochondrial, and plastid genomes were sequenced [5,6,7], enabling the development of genetic tools and DNA delivery methods, such as bacterial conjugation and microparticle bombardment [8,9]. Additional genetic tools for *T. pseudonana* include selectable markers [9,10], promoters [9], transformation vectors [9], inducible protein expression [9], RNA interference (RNAi) [11,12] and clustered regularly interspaced palindromic repeats/CRISPR-associated protein 9 (CRISPR/Cas9) [10,13,14]. Finally, methods for isolation of *T. pseudonana*’s chloroplast and mitochondria have been developed and proteomic data made available [15]. Most of the described genetic tools allow engineering of the *T. pseudonana* nuclear genome; however, engineering of its organelle genomes is still challenging. There are several advantages to engineering organelle genomes, including polycistronic gene organization, the lack of transgene silencing, reduced positional gene expression effects, and the compartmentalization of biosynthetic pathways, each of which simplifies engineering [16]. In preparation for exploiting these qualities, organelle genomes from multiple species have been cloned [17,18,19,20,21,22,23].

We recently demonstrated the cloning of the mitochondrial genome of *Phaeodactylum tricornutum*, a model diatom algae species, in baker’s yeast *Saccharomyces cerevisiae* and *Escherichia coli* [23]*. S. cerevisiae* has proven to be an excellent host for cloning large DNA fragments or whole genomes [19,24,25,26,27], and it was also demonstrated that chromosomes up to ~500 kbp could be cloned in *E. coli* [28]. To test the versatility and robustness of this method when applied to other algal species, we selected *T. pseudonana* because of the unique characteristics of its mitochondrial genome. First, the *T. pseudonana* mitochondrial genome is compact (~44 kbp), harboring a relatively small repeat region (~5 kbp) compared to the repeat region of *P. tricornutum* (~35 kbp). Second, *T. pseudonana* has a lower G+C content mitochondrial genome (30%) than *P. tricornutum* (35%). Third, *T. pseudonana*’s mitochondrial genome uses an alternative genetic code, which substitutes a typical stop codon (UGA) for a tryptophan residue [6]. This alternative genetic code could be beneficial during the development of a whole-genome delivery method as any engineered selection markers integrated in this genome would only function when delivered to the mitochondrial compartment, eliminating the need to screen against nuclear transformants [29].

Here, we report the successful cloning of *T. pseudonana*‘s mitochondrial genome in yeast and demonstrate that it can also be propagated in *E. coli*. In the first iteration (Design 1), the mitochondrial genome was cloned in its entirety (~44; ~58 kbp including plasmid backbone); in the second iteration (Design 2), ~3.8 kbp of the ~5 kbp repetitive region was excluded (~40; ~58 kbp including different plasmid backbone). Growth experiments performed on yeast in liquid media revealed that yeast strains carrying plasmids with cloned mitochondrial genomes had a slightly increased growth rate; however, after 24 hours, the yeast strains grew to the same (Design 1) or slightly lower (Design 2) end-point densities as control strains. When these genomes were propagated in *E. coli* on a low copy number plasmid, they had the same growth rate and end-point densities compared to the control strains. However, when grown with arabinose to increase the copy number of these genomes, all samples grew to significantly lower end-point densities after 11.5 h. Also, analysis of plasmids containing mitochondrial genomes following propagation in *E. coli* over 60 generations showed that about 17% of *T. pseudonana* mitochondrial genomes were mutated compared to 0% identified for equivalent experiments conducted using the *P. tricornutum* mitochondrial genome. Finally, RNA sequencing performed on *E. coli* harboring either alga’s mitochondrial genome found that expression can be detected for *T. pseudonana* and *P. tricornutum* mitochondrial genes.

## 2. Materials and Methods

### 2.1. Strains and Growth Conditions

*Thalassiosira pseudonana* (Culture Collection of Algae and Protozoa CCAP 1085/12) was grown in synthetic seawater (L1 medium) supplemented with 200 μM of sodium silicate (Na_2_SiO_3_-9H_2_0) (MP Biomedicals, Cat #: 191382, Solon, OH, USA) at 18 °C under cool white fluorescent lights (75 μE m^−2^ s^−1^) and a photoperiod of 16 h light: 8 h dark. L1 media was made as previously described [8]. Saccharomyces cerevisiae VL6−48 (ATCC MYA-3666: MATα his3-Δ200 trp1-Δ1 ura3-52 lys2 ade2-1 met14 cir^0^) was grown at 30 °C in rich yeast medium (2 × YPDA: 20 g L^−1^ yeast extract (BioShop Canada Inc., Cat #: YEX401.1, Burlington, ON, Canada), 40 g L^−1^ peptone (BioShop Canada Inc., Cat #: PEP403.1, Burlington, ON, Canada), 40 g L^−1^ glucose (BioShop Canada Inc., Cat #: GLU501.205, Burlington, ON, Canada), and 200 mg L^−1^ adenine hemisulfate (MilliporeSigma, Cat #: A2545, Darmstadt, Germany)), or synthetic complete medium lacking either histidine or both uracil and histidine (Teknova, Inc., Cat #: C7112 and C7221, respectively, Hollister, CA, USA). After yeast spheroplast transformation, all complete minimal media used contained 1 M sorbitol (BioShop Canada Inc., Cat #: SOR508.5, Burlington, ON, Canada) [27]. Escherichia coli Epi300 (Lucigen Corp., Cat #: LGN-EC300110, Middleton, WI, USA) was grown at 37 °C in Luria Broth (LB) or LB media supplemented with chloramphenicol (15 μg mL^−1^) (BioBasic Canada Inc., Cat #: CB0118, Markham, ON, Canada).

### 2.2. Genomic DNA Isolation by Modified Alkaline Lysis

DNA from *E. coli*, yeast, and algae was isolated as previously described [23].

### 2.3. DNA Fragment Preparation for Polymerase Chain Reaction (PCR) Cloning

#### 2.3.1. Design 1—Full Genome (pTP-PCR C1 and C2)

Cloning of mitochondrial genomes was performed using the method as described in Reference [23]. PCR amplification of mitochondrial fragments was performed using *T. pseudonana* genomic DNA. The mitochondrial genome was amplified as eight overlapping fragments (primers: P 1-4F/R and 6-8F/R, listed in Supplementary Table S1), as well as four additional fragments (primers: P 5F/R and 10-12F/R, listed in Supplementary Table S1) to amplify the *URA3* yeast selection marker and pPtGE31 plasmid backbone [30]. The pPtGE31 plasmid contains all the genetic elements required for selection and stable propagation in *S. cerevisiae*, *E. coli*, and *P. tricornutum*. In addition, this plasmid contains an origin of transfer to allow for plasmid transfer using bacterial conjugation. All primers were manually designed. Forward and reverse primers for fragments 2–3 and 7–8, as well as the reverse primer of fragment 1, were designed to be 40 bp in length. Primers 60 bp in length were designed for fragments 4–6 and 9–12, as well as the forward primer of fragment 1. Overlapping homology between fragments was between 80 and 635 bp to allow for efficient yeast assembly.

Each fragment was individually amplified in a 50 μL PCR reaction using 1 μL of PrimeSTAR GXL polymerase (Takara Bio Inc., Cat #: R050A, Kusatsu, Shiga, Japan), 1 μL of template DNA (1–100 ng μL^−1^ genomic algal DNA or plasmid DNA isolated from *E. coli*), and the respective forward and reverse primers each at a final concentration of 0.2 μM. The thermocycler (Bio-Rad Laboratories, Inc., Cat #: 1861096, Hercules, CA, USA) conditions for fragments 2, 4–9, and 11–12 were as follows: 25 cycles of 98 °C for 10 s, 60 °C for 15 s, and 68 °C for 600 s, and one cycle of 68 °C for 600 s, finishing with an infinite hold at 12 °C. The thermocycler for fragment 1 was programmed as follows: 5 cycles of 98 °C for 10 s, 50 °C for 15 s, and 68 °C for 420 s, followed by 20 cycles of 98 °C for 10 s, 60 °C for 15 s, and 68 °C for 420 s, and one cycle of 68 °C for 600 s, finishing with an infinite hold at 12 °C. The thermocycler for fragment 3 was programmed as follows: 25 cycles of 98 °C for 10 s, 50 °C for 15 s, and 68 °C for 660 s, and one cycle of 68 °C for 600 s, finishing with an infinite hold at 12 °C. The thermocycler for fragment 10 was programmed as follows: 25 cycles of 98 °C for 10 s, 55 °C for 15 s, and 68 °C for 60 s, and one cycle of 68 °C for 600 s, finishing with an infinite hold at 12 °C. PCR product amplification was confirmed by performing agarose gel electrophoresis with 2 μL of PCR product on a 1.4% agarose (*w*/*v*) gel. To eliminate plasmid template DNA, PCR products were treated with DpnI restriction enzyme as described in Reference [23].

#### 2.3.2. Design 2—Reduced Genome Lacking the Repetitive Region (pTP-PCR C3 and C4)

PCR amplification of mitochondrial fragments was performed using isolated *T. pseudonana* genomic DNA as the template DNA. The mitochondrial genome was amplified as seven overlapping fragments (primers: P 1R, 2-3F/R, 4F, 6R, 8R, 13-14F/R, and 17-18F, listed in Supplementary Table S1), as well as two additional fragments (primers: P15-16F/R, listed in Supplementary Table S1) to amplify the pAGE3.0 plasmid with homology to sequence directly flanking the repeat region. The pAGE3.0 plasmid is a derivation of pPtGE31 providing additional elements for selection and stable propagation in *Sinorhizobium meliloti* [31]. All primers were manually designed. Forward and reverse primers for fragments 1–2, 5, as well as the forward primer for fragments 4 and 6, and the reverse primer for fragments 3 and 9 were designed to be 40 bp in length. Primers 60 bp in length were designed for the forward primer of fragment 3 and the reverse primer of fragment 4. The reverse primer of fragment 6, the forward primer of fragment 9, and the primers of fragments 7–8 were 80 bp in length. Overlapping homology between fragments were between 80 and 635 bp to allow for efficient yeast assembly.

Each fragment was individually amplified in a 50 μL PCR reaction using 1 μL of PrimeSTAR GXL, 1 μL of template DNA (1–100 ng μL^−1^ genomic DNA or plasmid DNA isolated from *E. coli*), and the respective forward and reverse primers each at a final concentration of 0.2 μM. The thermocycler for fragments 1, 3–5, and 7–9 was programmed as follows: 25 cycles of 98 °C for 10 s, 60 °C for 15 s, and 68 °C for 600 s, and one cycle of 68 °C for 600 s, finishing with an infinite hold at 12 °C. The thermocycler for fragments 2 and 6 was programmed as follows: 30 cycles of 98 °C for 10 s, 50 °C for 15 s, and 68 °C for 660 s, followed by one cycle of 68 °C for 600 s, finishing with an infinite hold at 12 °C. PCR product amplification was confirmed by performing agarose gel electrophoresis with 2 μL of PCR product on a 1.4% agarose (*w*/*v*) gel. To eliminate plasmid template DNA, PCR products were treated with DpnI restriction enzyme as described in Reference [23].

### 2.4. Yeast Spheroplast Transformation Protocol

Yeast spheroplast transformation was performed as previously described in Reference [23].

### 2.5. E. coli Transformation

*E. coli* transformation was performed as previously described in Reference [23].

### 2.6. Screening Strategy

#### 2.6.1. Screening Yeast Colonies

To identify positive clones, individual yeast colonies were screened as previously described in Reference [23], using selective 2% agar plates either lacking both histidine and uracil for pTP-PCR C1/2, or histidine for pTP-PCR C3/4. Multiplex primers used for screening yeast colonies differ from the original protocol and are listed in Supplementary Table S1.

#### 2.6.2. Screening *E. coli* Colonies

To identify positive clones, individual *E. coli* colonies were screened as previously described in Reference [23] with the following modifications to the restriction enzyme digestion reactions. For pTP-PCR C1.1/2.1, digestion reactions were generated using 7 μL of DNA (~5000 ng μL^−1^), 2 μL of NEBuffer™ 3.1 restriction buffer (New England Biolabs Ltd., Cat #: B7203S, Ipswich, MA, USA), 0.4 μL PvuI (New England Biolabs Ltd., Cat #: R0150S, Ipswich, MA, USA), and 10.6 μL of water. For pTP-PCR C3.1/4.1, digestion reactions were generated using 7 μL of DNA (~5000 ng μL^−1^), 2 μL of CutSmart^®^ Buffer (New England Biolabs Ltd., Cat #: B7204S, Ipswich, MA, USA), 0.4 μL PmeI (New England Biolabs Ltd., Cat #: R0560S, Ipswich, MA, USA), 0.4 μL BamHI-HF (New England Biolabs Ltd., Cat #: R3136S, Ipswich, MA, USA), and 10.2 μL of water. Reaction mixtures were transferred to a Bio-Rad thermocycler and incubated either at 37 °C for 90 min, or 37 °C for 90 min followed by 65 °C for 20 min for pTP-PCR C1.1/2.1 and C3.1/4.1, respectively. After confirmation by multiplex PCR and diagnostic restriction enzyme digestion, the four plasmids underwent next-generation whole plasmid sequencing at CCIB DNA Core (Massachusetts General Hospital, Boston, MA, USA).

### 2.7. Evaluation of Growth Phenotypes of Host Strains

#### 2.7.1. *S. cerevisiae* Growth in Liquid Media

Growth rates were evaluated for *S. cerevisiae* strains harboring pTP-PCR C1, C2, C3, and C4 plasmids, or pPtGE31 and pAGE3.0 control plasmids (lacking mitochondrial genome), as described in Reference [23], with absorbance (A_600_) measurements taken every 15 min for 24 h. This experiment was performed with three biological replicates, each with four technical replicates, therefore, 12 readings were obtained and averaged for each strain, the standard error of the mean was calculated, and the curves were plotted. End-point densities at 1440 min were averaged for each strain and the standard error of the mean was calculated. The doubling time (td) of each replicate was determined using the R package Growthcurver (Sprouffske K., Growthcurver, http://github.com/sprouffske/growthcurver, 2016) [32]. The td was averaged from the 12 replicates for each strain, and the standard error of the mean was calculated.

#### 2.7.2. *E. coli* Growth in Liquid Media 

Growth rates were evaluated for *E. coli* strains harboring pTP-PCR C1.1, C2.1, C3.1, and C4.1 plasmids or pPtGE31 and pAGE3.0 control plasmids (lacking mitochondrial genome), as described in Reference [23], except the samples were not placed on ice and A_600_ measurements were taken every 15 min for 11.5 h. This experiment was performed with three biological replicates, each with four technical replicates, therefore, 12 readings were obtained and averaged for each strain, the standard error of the mean was calculated, and the curves were plotted. End-point densities at 705 min were averaged for each strain and the standard error of the mean was calculated. The doubling time (td) of each replicate was determined using the R package Growthcurver (Sprouffske K., Growthcurver, http://github.com/sprouffske/growthcurver, 2016) [32]. The td was averaged from the 12 replicates for each strain and the standard error of the mean was calculated. Replicates shown to be major outliers were removed from the dataset (Supplementary Note S1).

### 2.8. Bacterial RNA Extraction

*E. coli* harboring the pTP-PCR C2.1, pPT-PCR C2.1 [23], or pPtGE31 plasmid (lacking mitochondrial genome), were inoculated overnight in LB media supplemented with chloramphenicol (15 μg mL^−1^) from frozen strain stocks. In the morning, 1 mL of cells was diluted into 25 mL of LB media supplemented with chloramphenicol (15 μg mL^−1^), and grown for 120 min at 37 °C until optical density (OD_600_) reached 1. Subsequently, the RNA stabilization of the culture was performed using RNAprotect Bacteria Reagent (Qiagen, Inc., Cat #: 76506, Hilden, Germany). Briefly, 400 μL (2 × 10^8^ cells) of culture was transferred to a 15 mL Falcon tube containing 800 μL of RNAprotect Bacteria Reagent, and the suspension was vortexed for 5 s and incubated at room temperature (RT) for 5 min. Total RNA was isolated using the RNeasy Mini Kit (Qiagen, Inc., Cat #74104, Hilden, Germany) according to the manufacturer’s instructions. Following treatment with DNase using RNase-free DNase Set (Qiagen Inc., Cat # 79254, Hilden, Germany), the RNA concentration was determined using DeNovix (DeNovix Inc., Wilmington, DE, USA) and the integrity was verified by running 400 ng of RNA on a 1% agarose (*w*/*v*) gel. RNA samples were stored at −80 °C until use.

### 2.9. RNA Sequencing

The quality of isolated RNA (Methods Section 2.8) was validated using the Agilent Bioanalyzer 2100 (Agilent Technologies, Inc., Santa Clara, CA, USA). The RNA library was created and sequenced using the NextSeq 550 platform (single end 150 mid-output), with rRNA depleted using the NEB bacterial rRNA depletion kit (E7850S). Read quality was evaluated using FastQC v0.11.9, and reads were trimmed using Trimmomatic v0.39 in single-end mode using the parameters AVGQUAL:25 CROP:150 MINLEN:100 [33,34,35]. Reads for the pTP-PCR C2.1 and pPT-PCR C2.1 strains were mapped against their respective mitochondrial plasmid maps using bowtie 2.26 in single-end mode with the parameters no-unal -k 1. Read counts were generated using htseq-count. 

### 2.10. Plasmid Stability Assay

#### 2.10.1. Propagation of *E. coli* Strains

*E. coli* harboring either pTP-PCR C2.1 or pPT-TAR C1 [23] plasmids were inoculated from frozen strain stocks (note that these stocks were generated by transferring cloned mitochondrial DNA from yeast to *E. coli*, isolating single colonies on LB plates, and then grown overnight in liquid before freezing) overnight at 37 °C (225 rpm) in 5 mL of LB media supplemented with chloramphenicol (15 μg mL^−1^). The saturated overnight culture was adjusted to an OD_600_ of 0.1 and frozen strain stocks were generated (we defined this as G0). Next, 1 μL of the adjusted culture was added to 50 mL of LB media supplemented with chloramphenicol (15 μg mL^−1^). Cultures were grown at 37 °C (225 rpm) to approximately an OD_600_ of 3. Four repetitions (~60 generations) of diluting grown cultures to an OD_600_ of 0.1 and passaging 1 μL of adjusted culture into 50 mL of LB media supplemented with chloramphenicol (15 μg mL^−1^) were performed. After four serial passages, frozen strain stocks of each bacterial strain adjusted to an OD_600_ of 0.1 were generated for analysis of plasmid stability (G60).

#### 2.10.2. Analysis of Descendant *E. coli* Colonies

Strain stocks of pTP-PCR C2.1 or pPT-TAR C1 from the start (G0) and end (G60) of propagation were thawed on ice for 20 min and then diluted 1:5000 with LB media in 1.5 mL microcentrifuge tubes. Next, 100 μL of each diluted culture was plated separately onto selective LB agar plates supplemented with chloramphenicol (15 μg mL^−1^) and grown at 37 °C for 24 h to obtain single colonies. Thirty single colonies of each construct were struck onto selective LB agar plates supplemented with chloramphenicol (15 μg mL^−1^) and grown for 12 h at 37 °C. Next, each streak was resuspended in a PCR tube containing 100 μL of TE buffer. The cell mixture was transferred to a Bio-Rad thermocycler and incubated at 95 °C for 15 min to lyse the cells. Using a microfuge, the cellular debris was pelleted to the bottom of the PCR tube and 1 μL of supernatant was used as a template for diagnostic multiplex PCR. Two additional diagnostic primer sets (P 19-20F/R, listed in Supplementary Table S1) were generated for this plasmid stability analysis. Primer pairs P 19-20F/R were designed to have an optimized melting temperature of 60 °C using the online tool Primer3 (http://bioinfo.ut.ee/primer3-0.4.0/). To test plasmid stability, primer sets P 19-24F/R were used to generate 140, 229, 300, 440, 540, and 606 bp amplicons, respectively. Then, 2 μL of the PCR products were loaded onto a 2% agarose (*w*/*v*) gel for electrophoresis and analyzed.

### 2.11. Statistical Analyses

Statistical analyses were performed using Microsoft Excel spreadsheet software. Pair-wise comparisons were made using a Student’s *t*-test with either equal or unequal variance based on the result of an F-test. The error bar shown represents the standard error of the mean.

## 3. Results

### 3.1. Cloning of T. pseudonana Mitochondrial Genomes

**Figure 1:**
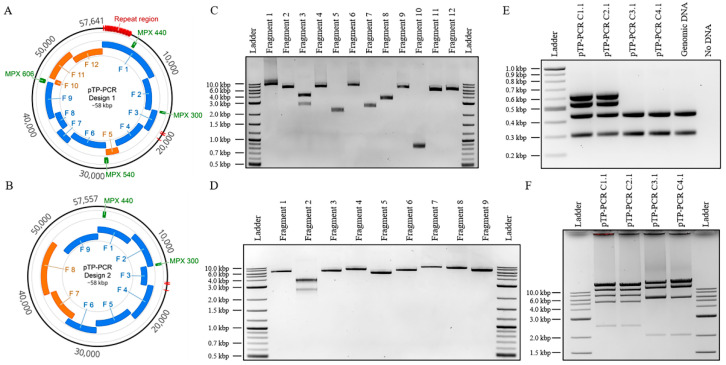
Design, amplification, and analysis of cloned *T. pseudonana* mitochondrial genomes. (**A**,**B**) Plasmid maps of *T. pseudonana* mitochondrial genomes cloned with the repeat region (A—Design 1) or without (B—Design 2). The relative sizes and positions of the mitochondrial genome fragments (blue) and plasmid backbone fragments (orange) are shown. In addition, the four multiplex polymerase chain reaction (PCR) amplicons used for diagnostic screening and their sizes in bp are indicated (green). These images were generated using Geneious version 2020.2.4, created by Biomatters. (**C**) Agarose gel electrophoresis of the 12 PCR-amplified fragments used to assemble plasmids as specified in Design 1. The resulting amplicon sizes for fragments 1 to 12 are 10,735, 6092, 3610, 6274, 2152, 7035, 2512, 3250, 6216, 859, 5367, and 5870 bp, respectively. Note: for fragment 3, there was a nonspecific amplicon; however, it did not prevent the correct assembly. (**D**) Agarose gel electrophoresis of the nine PCR-amplified fragments used to assemble plasmids as specified in Design 2. The resulting amplicon sizes for fragments 1 to 9 are 6092, 3610, 6254, 7174, 5417, 6372, 9136, 8441, and 6810 bp, respectively. Note: for fragment 2, there was a nonspecific amplicon; however, it did not prevent the correct assembly. (**E**) Multiplex PCR screen of four cloned algal mitochondrial genomes isolated from *E. coli* with expected amplicon sizes of: 300, 440, 540, and 606 bp. Note: Multiplex amplicons 540 and 606 bp can only be amplified for Design 1 genomes. (**F**) Diagnostic restriction digest of the four cloned algal mitochondrial genomes. For Design 1 genomes (pTP-PCR C1.1/2.1), after PvuI restriction enzyme digestion, the expected band sizes are 6, 2454, 4862, 6262, 12,903, 15,405, and 15,749 bp. For Design 2 genomes (pTP-PCR C3.1/4.1), after PmeI and BamHI restriction enzymes digestion, expected band sizes are 2031, 5693, 12,012, 16,721, and 20,960 bp.

Using a PCR-based approach, we cloned *T. pseudonana*’s mitochondrial genome in its entirety (Design 1: 57,641 bp; composed of a 43,827 bp mitochondrial genome, 11,742 bp plasmid backbone, and 2072 bp *URA3* additional selection marker for yeast) as well as a reduced version lacking 3.8 kbp of the ~5.0 kbp repetitive sequence (Design 2: 57,557 bp; composed of a 40,034 bp mitochondrial genome and a 17,523 bp plasmid backbone) (Figure 1). For Design 1, the complete genome was PCR-amplified in eight overlapping fragments from total *T. pseudonana* DNA. Four additional overlapping fragments were amplified, including the *URA3* yeast selection marker (F5) and the pPtGE31 plasmid [30] backbone (F10–12), which contains all elements required for plasmid propagation in yeast and *E. coli*. In total, 12 DNA fragments were amplified (Figure 1C) and assembled following transformation and homologous recombination in *S. cerevisiae*, yielding 187 yeast colonies (Table 1). For Design 2, the genome was PCR-amplified in seven overlapping fragments that excluded a 3.8 kbp repeat region. The pAGE3.0 plasmid [31] backbone was amplified as two additional fragments (F7–8) to provide all the elements required for propagation in yeast and *E. coli*. In total, nine DNA fragments were amplified (Figure 1D) and assembled in yeast, yielding 680 colonies (Table 1). For each design of the mitochondrial genome, two clones identified to be correct in yeast by diagnostic multiplex PCR were selected and transformed into *E. coli*. After moving the assembled plasmids to *E. coli*, they were validated by diagnostic multiplex PCR and restriction enzyme digest (Figure 1E, F). For Design 1, the two selected clones were named pTP-PCR C1.1/C2.1, and, for Design 2, pTP-PCR C3.1/C4.1. All four clones were sequenced and analyzed for mutations. 

### 3.2. Sequence Analysis of Cloned T. pseudonana Mitochondrial Genomes

Sequences obtained for the pTP-PCR plasmids were aligned to reference sequences (Supplemental Files S2 and S3). Upon analyzing mutations, pTP-PCR C1.1, C2.1, C3.1, and C4.1 had an average of 18 changes per mitochondrial genome (Table 2, Supplementary Table S2). We observed approximately twice the number of mutations in clones for Design 1; however, most of these mutations mapped to the repetitive region (Supplementary Table S2), which could be due to sequencing errors. Mutations could have occurred during the cloning process (PCR amplification of fragments) or propagation in the host organisms. It is also plausible that some of these variants could naturally exist in the heterogeneous population of *T. pseudonana* mitochondrial genomes, or variations between our strain and the sequenced genome. If desired, individual fragments could be cloned and confirmed by sequencing before use in yeast assembly.

### 3.3. Maintenance of T. pseudonana Mitochondrial Genomes in Host Organisms

**Figure 2:**
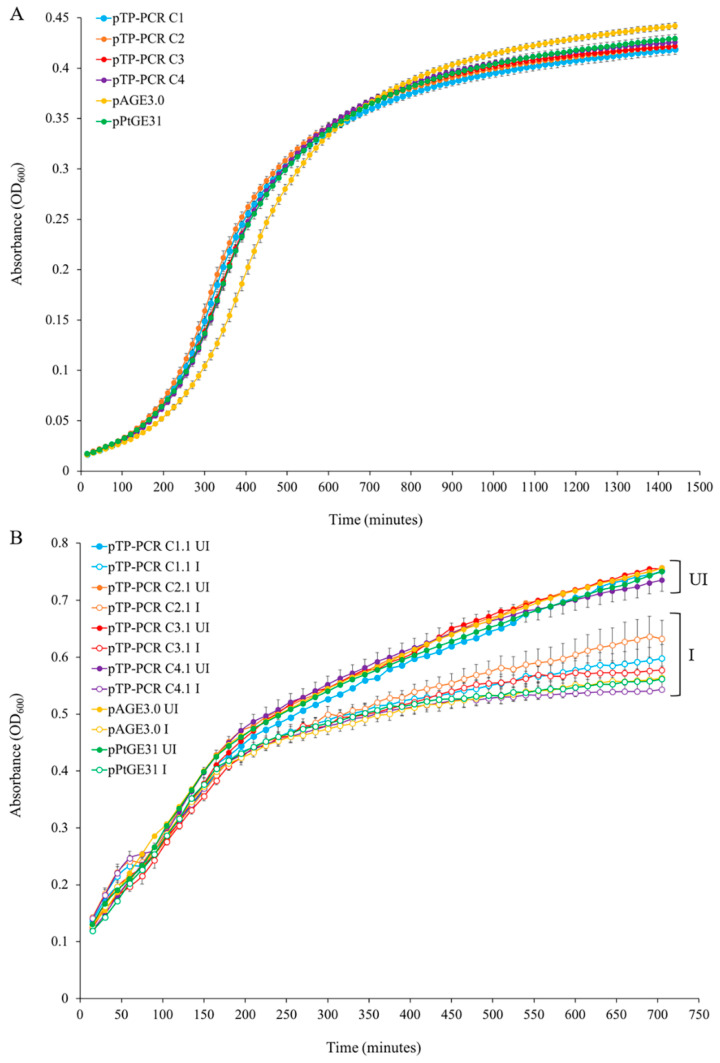
Growth of host strains harboring cloned *T. pseudonana* mitochondrial genomes in liquid media. (**A**) Growth curves of *S. cerevisiae* strains grown in liquid synthetic complete media lacking histidine. (**B**) Growth curves of *E. coli* strains grown in liquid Luria-Bertani (LB) media supplemented with chloramphenicol only (UI—un-induced) or with chloramphenicol and arabinose (I—induced). Each time point is the average of three biological replicates, each with four technical replicates, and error bars representing standard error of the mean.

**Figure 3:**
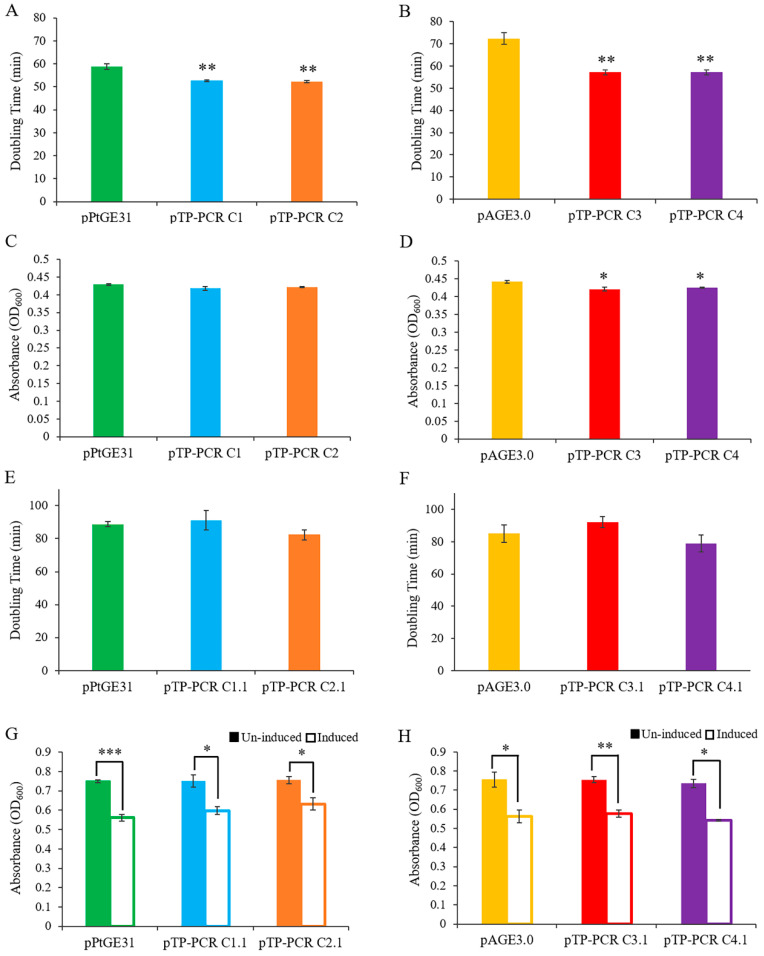
Growth phenotypes of *S. cerevisiae* and *E. coli* harboring a cloned *T. pseudonana* mitochondrial genome. The growth rate of *S. cerevisiae* harboring the full (**A**) and reduced (**B**) mitochondrial genome of *T. pseudonana* compared to control plasmids pPtGE31 and pAGE3.0, respectively. The maximum cell density reached by *S. cerevisiae* harboring the full (**C**) and reduced (**D**) mitochondrial genome compared to control plasmids. The growth rate of *E. coli* harboring the full (**E**) and reduced (**F**) mitochondrial genome, compared to control plasmids (uninduced conditions). Maximum cell density reached by *E. coli* harboring the full (**G**) and reduced (**H**) mitochondrial genome compared to control plasmids. Maximum density was compared in un-induced and arabinose induced conditions. Note: Solid bar represents un-induced and outlined bar represents induced conditions. Three biological replicates, each with four technical replicates, were used for data analysis. The scores represent means ± standard error of three biological replicates. Asterisks represent a significant difference from control plasmid (**A**–**F**), and/or between un-induced and induced *E. coli* harboring the same plasmid (**G**–**H**) (Student’s *t*-test: * *p* < 0.05, ** *p* < 0.01; *** *p* < 0.001).

We sought to examine the burden of propagating the cloned mitochondrial genomes in eukaryotic and prokaryotic host strains. *S. cerevisiae* and *E. coli* were used as host organisms to clone and store the *T. pseudonana* mitochondrial genome. We measured the growth of *E. coli* and yeast strains in liquid media using a 96-well plate reader. Growth experiments performed for yeast revealed that strains carrying plasmids with cloned mitochondrial genomes had a slightly increased growth rate; however, after 24 h, the yeast strains grew to the same (Design 1) or slightly lower (Design 2) end-point densities as compared to control strains (Figure 2A and Figure 3A–D). For propagation in *E. coli*, we tested conditions where plasmids with mitochondrial genomes were maintained either as low or high (induced with arabinose) copy number. When these genomes were propagated in *E. coli* without induction of plasmid copy number, there was no significant difference in the growth rate as compared to the control strain (Figure 2B and Figure 3E,F). When grown with arabinose, all samples grew to a significantly lower end-point densities than the uninduced strains (Figure 2B and Figure 3G–H); however, there were no significant differences between the control plasmid and plasmids harboring a mitochondrial genome within each growth condition (Figure 3G,H).

Additionally, when propagated in *E. coli* for an extended time (>50 generations), we observed that a small fraction of genomes were being mutated, as was evident by an absent PCR amplicon when clones were evaluated with multiplex PCR (data not shown). To further investigate this, we evaluated one cloned mitochondrial genome (pTP-PCR C2.1) directly after transferring from yeast to *E. coli* (“G0”) or after approximately 60 generations (“G60”). Since we did not observe similar mutations in our previous work cloning the *P. tricornutum* mitochondrial genome, we used our cloned *P. tricornutum* mitochondrial genome (pPT-TAR C1) as a control [23]. In total, 30 colonies for both *T. pseudonana* (from clone pTP-PCR C2.1) and *P. tricornutum* (from clone pPT-TAR C1) were evaluated at G0 and G60 using a higher resolution multiplex PCR screen with six amplicons.

**Figure 4:**
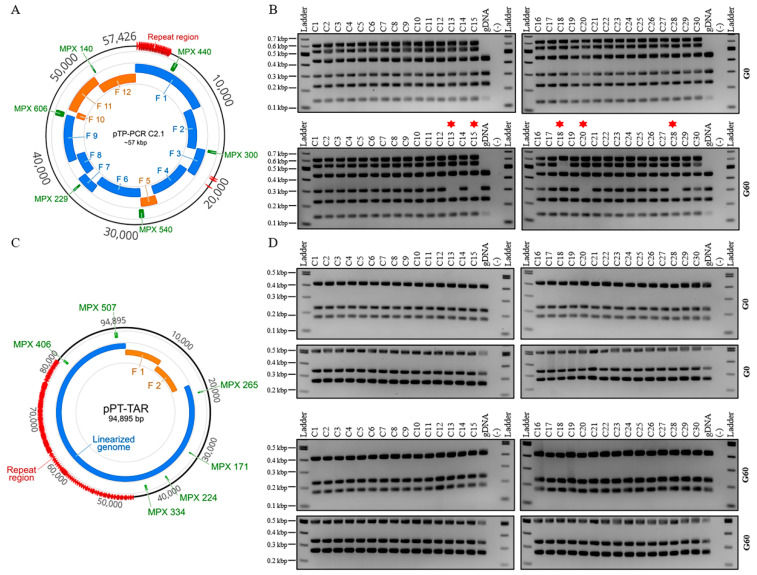
Plasmid stability assay of cloned *T. pseudonana* and *P. tricornutum* mitochondrial genomes over 60 generations. Thirty single colonies of either pTP-PCR C2.1 (**A**,**B**) or pPT-TAR C1 (**C**,**D**) were assayed by multiplex PCR after transfer from yeast to *E. coli* (G0), and after approximately 60 generations (G60) in liquid LB media supplemented with chloramphenicol (15 μg mL^−1^). Notes: 1—in (B), (G60) colonies 13, 15, 18, 20, and 28 (red asterisk) are missing one PCR amplicon; 2—for *T. pseudonana* genomic DNA (gDNA), only the three fragments were expected to amplify, 229, 300, and 440 bp, but a small nonspecific band is also visible around 150 bp.

At G0, all 30 *E. coli* clones harboring either plasmid showed successful amplification of all six amplicons. At G60, all 30 *E. coli* clones harboring pPT-TAR C1 had a complete genome as analyzed by multiplex PCR, suggesting that, over 60 generations, this plasmid is stably maintained. However, only 25 of 30 *E. coli* clones containing pTP-PCR C2.1 had complete genomes at G60, as analyzed by multiplex PCR (Figure 4). These 5 clones were further analyzed by restriction enzyme digest (Supplementary Figure S1). Four of these plasmids showed aberrant restriction enzyme banding patterns, suggesting a deletion or rearrangement. Three of these plasmids were sequenced, which confirmed that the absent multiplex amplicon resulted from deletion events (Supplementary Figure S2).

### 3.4. Assessing the Expression of T. pseudonana and P. tricornutum Mitochondrial Genes in E. coli

RNA expression of mitochondrial genes was confirmed for pTP-PCR C2.1 and pPT-PCR C2.1 in *E. coli* with three biological replicates. Read counts were compared against pPtGE31 (lacking the mitochondrial genome). As expected, no reads from pPtGE31 mapped against the algal mitochondrial genomes, while genes from the pTP-PCR C2.1 and pPT-PCR C2.1 samples showed low read coverage. Low coverage of the mitochondrial genomes was likely obtained because the rRNA depletion kit selected failed to deplete the *E. coli* host’s rRNA. The bacterial selection marker *CAT* (providing resistance to chloramphenicol) was detected in all samples as expected; however, low read coverage was obtained for many of the genes that are on the pTP-PCR C2.1 and pPT-PCR C2.1 mitochondrial genomes (Supplementary Tables S3 and S4). The genes with the most mapped reads from the mitochondrial genome plasmids were rRNA and *cox1* genes. Although low read coverage was obtained, the counts confirm expression of many of the mitochondrial genes from plasmids propagated in the *E. coli* hosts.

## 4. Discussion

The biotechnological potential of organelle engineering is constrained by the lack of reliable methods to clone and deliver organelle genomes to the corresponding compartment. Towards this goal, we have previously developed a method for cloning and manipulating *P. tricornutum* mitochondrial genomes in host organisms [23]. Here, we demonstrated that this method could be adapted to another microalga, *T. pseudonana*. As for *P. tricornutum*, we observed similar growth rates between host strains carrying empty plasmids and those with cloned *T. pseudonana* mitochondrial genomes. RNA expression analysis showed that most of the mitochondrial genes from *P. tricornutum* and *T. pseudonana* plasmids are expressed in *E. coli*. Interestingly, mitochondrial gene expression in the host did not affect genome stability for *P. tricornutum* mitochondrial genomes. Plasmids with *T. pseudonana* genomes were less stable with continued propagation in *E. coli*; after 60 generations, 17% of genomes were mutated.

It has been observed previously that cloning low G+C-content DNA into bacteria can be problematic. As the G+C-content decreases, the probability of any sequence producing a spontaneous promoter or origin of replication becomes more likely, which can result in plasmid toxicity and instability [36]. The addition of a second origin of replication can stall the replication fork, leading to plasmid rearrangements [37]. Challenges arise with G+C-contents as low as 35–40%; however, it was shown that this DNA could be stably maintained at a low copy number [36]. Additionally, it has been shown that by engineering the vector backbone to be more accommodating, low G+C-content genomes such as *Lactobacillus helveticus* (35%) could be cloned [36]. Future investigation could focus on optimizing the pTP-PCR plasmids for stability.

Although we can confirm that most of the mitochondrial genes on the pTP-PCR C2.1 and pPT-PCR C2.1 plasmids are expressed in *E*. *coli* hosts, the low read coverage obtained prevented us from performing a reliable differential expression analysis. However, the data does demonstrate that genes of the algal mitochondrial genomes are expressed in the *E.* *coli* hosts. To determine what genes of the mitochondrial genomes are more strongly expressed in the *E. coli* hosts, we will need to obtain higher coverage of the mitochondrial genome by successfully depleting the host’s rRNA in a future experiment.

Now that two algal mitochondrial genomes have been cloned in host strains, we have a better understanding of potential hurdles that can be encountered when applying this method to other species. As the next step, a robust method for delivery of these genomes to mitochondria will need to be developed. First, the mitochondrial genomes will need to be engineered with mitochondria-specific selectable markers. Using antibiotics that target organelle-specific processes, previous studies have demonstrated an increased efficacy of antibiotic resistance proteins when they are localized to the organelle compartment [38,39]. Further, there are two promising antibiotic selection markers, zeocin and chloramphenicol, which have been described for use in the mitochondria of *Chlamydomonas reinhardtii* [40] and the chloroplast of *P. tricornutum* [41], respectively. Expression of the antibiotic resistance gene can be biased towards the mitochondria by using mitochondrial promoter and terminator sequences. Additionally, any selection markers generated for *T. pseudonana*’s mitochondria will be designed using alternative genetic code (UGA will be used for a tryptophan instead of a stop codon), allowing antibiotic resistance proteins to be expressed only in the mitochondrial compartment. These two design features will generate a powerful system for developing the genetic tools required for mitochondrial DNA delivery in *T. pseudonana*.

## 5. Conclusions

We have demonstrated that a previously developed method for cloning and manipulating mitochondrial genomes can be applied to additional microalga. With a PCR-based approach, we cloned the mitochondrial genome of *T. pseudonana* in its entirety (~44 and; ~58 kbp including plasmid backbone) or lacking a repetitive region (~40; ~58 kbp including different plasmid backbone). The cloned genomes imposed no substantial growth burden on *S. cerevisiae* and *E. coli* when these host organisms were used to propagate the plasmids. In *E. coli*, some plasmid instability was observed after 60 generations, likely attributable to the low G+C-content of the mitochondrial genome. RNA sequencing was performed, and it was found that mitochondrial genes were being expressed from the plasmids harbored in *E. coli*. In this study, we replicated the previous methods for cloning and manipulating algal mitochondrial genomes using *T. pseudonana*. Subsequent work will focus on developing the technologies required for efficient mitochondrial DNA delivery.

## Figures and Tables

**Table 1:** Cloning of the *T. pseudonana* full and reduced mitochondrial genomes in the host organisms *S. cerevisiae* and *E. coli*. Two PCR-cloning assemblies were performed in *S. cerevisiae*. Correct genomes identified by multiplex PCR were subsequently transformed into *E. coli*. The diagnostic multiplex PCR was repeated on *E. coli* clones, and final genomes selected. For the *E. coli* media, CM indicates chloramphenicol antibiotic. Four-amplicon multiplex PCR as shown in Figure 1E were used.

Design Assembly Type DNA Source	*S. cerevisiae*			*E. coli*			
	Media	Colony Count	Multiplex PCR Screen Positive/Total	Media	Selected Yeast Colony: *E. coli* Colony Count	Multiplex PCR Screen Positive/Total	Final Genomes Names Selected for Analysis
1—Full Genome PCR—12 Fragments Genomic DNA	-Histidine -Uracil	187	15/20	CM	C1: 11 C2: 1137	C1 = 8/8 C2 = 4/4	pTP-PCR C1.1 pTP-PCR C2.1
2—Reduced Genome PCR—9 Fragments Genomic DNA	-Histidine	680	18/20	CM	C1: 4366 C2: 3530	C1 = 5/5 C2 = 5/5	pTP-PCR C3.1 pTP-PCR C4.1

**Table 2:** Summary of mutations identified in the cloned *T. pesudonana* mitochondrial genomes. Identified mutations are categorized as point mutations (synonymous, missense, nonsense, and those found in non-coding regions) or gap mutations (insertions and deletions, either non-coding or coding).

Clone	Point Mutations				Gap Mutations				Total
	Synonymous	Missense	Nonsense	Non-Coding	Non-Coding		Coding		
					Insertion	Deletion	Insertion	Deletion	
pTP-PCR C1.1	1	6	0	8	0	6	0	3	24
pTP-PCR C2.1	1	3	0	7	2	8	0	2	23
pTP-PCR C3.1	0	5	0	0	0	1	2	4	12
pTP-PCR C4.1	1	5	0	3	0	1	0	2	12

## Supplementary Materials

The following are available online at https://www.mdpi.com/2079-7737/9/11/358/s1, in Supplemental File 1: Figure S1: PvuI restriction digest analysis of mutated mitochondrial genomes from the plasmid stability assay, Figure S2: Sequencing analysis of mutated mitochondrial genomes from the plasmid stability assay, Table S1: Primers used in the cloning and screening of all *T. pseudonana* mitochondrial genomes cloned, Table S2: List of mutations identified in cloned *T. pseudonana* mitochondrial genomes by next-generation sequencing, Table S3: Count of raw RNA sequencing reads for strains with either the pPT-PCR C2.1 genomes or plasmid backbone alone (pPtGE31), Table S4: Count of raw RNA sequencing reads for strains with either the pTP-PCR C2.1 genome or plasmid backbone alone (pPtGE31), Note S1: Determination of outliers in the calculation of doubling time for *E. coli* strains. In addition two genbank files (Supplemental Files S2 and S3) with sequence information for pTP-PCR Design 1 and pTP-PCR Design 2 respectively are also available.
