# Supplementary material for: Cloning of Thalassiosira pseudonana’s Mitochondrial Genome in Saccharomyces cerevisiae and Escherichia coli
Source: Biology (Basel). 2020 Oct 26;9(11):358. doi: 10.3390/biology9110358 (PMC7693118; doi:10.3390/biology9110358)
Supplement: Supplementary file 1 [file biology-09-00358-s001.zip › biology-962175-supplementary-English.pdf]

Cloning of *Thalassiosira pseudonana*'s mitochondrial genome in *Saccharomyces cerevisiae* and *Escherichia coli*

Ryan R. Cochrane[1], Stephanie L. Brumwell[1], Arina Shrestha[1], Daniel J. Giguere[1], Samir Hamadache[1], Gregory B. Gloor[1], David R. Edgell[1], and Bogumil J. Karas[1*].

[1]Department of Biochemistry, Schulich School of Medicine and Dentistry, The University of Western Ontario, London, N6A 5C1, Ontario, Canada

*Correspondence: bkaras@uwo.ca

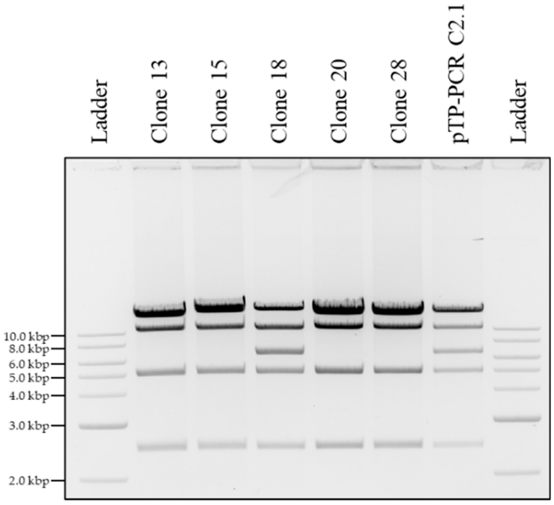

Supplementary Figure S1. PvuI restriction digest of five mutated pTP-PCR C2.1 clones from the plasmid stability assay (Figure 4) and pTP-PCR C2.1 (control). Band sizes of 6, 2454, 4863, 6262, 12869, 15404, and 15749 bp are expected. Notes: 1 – Mutated pTP-PCR C2.1 clones 13, 15, 20, and 28 show incorrect restriction pattern confirming the result from the multiplex PCR experiment; 2 - The 6 bp band is not visible in this gel.

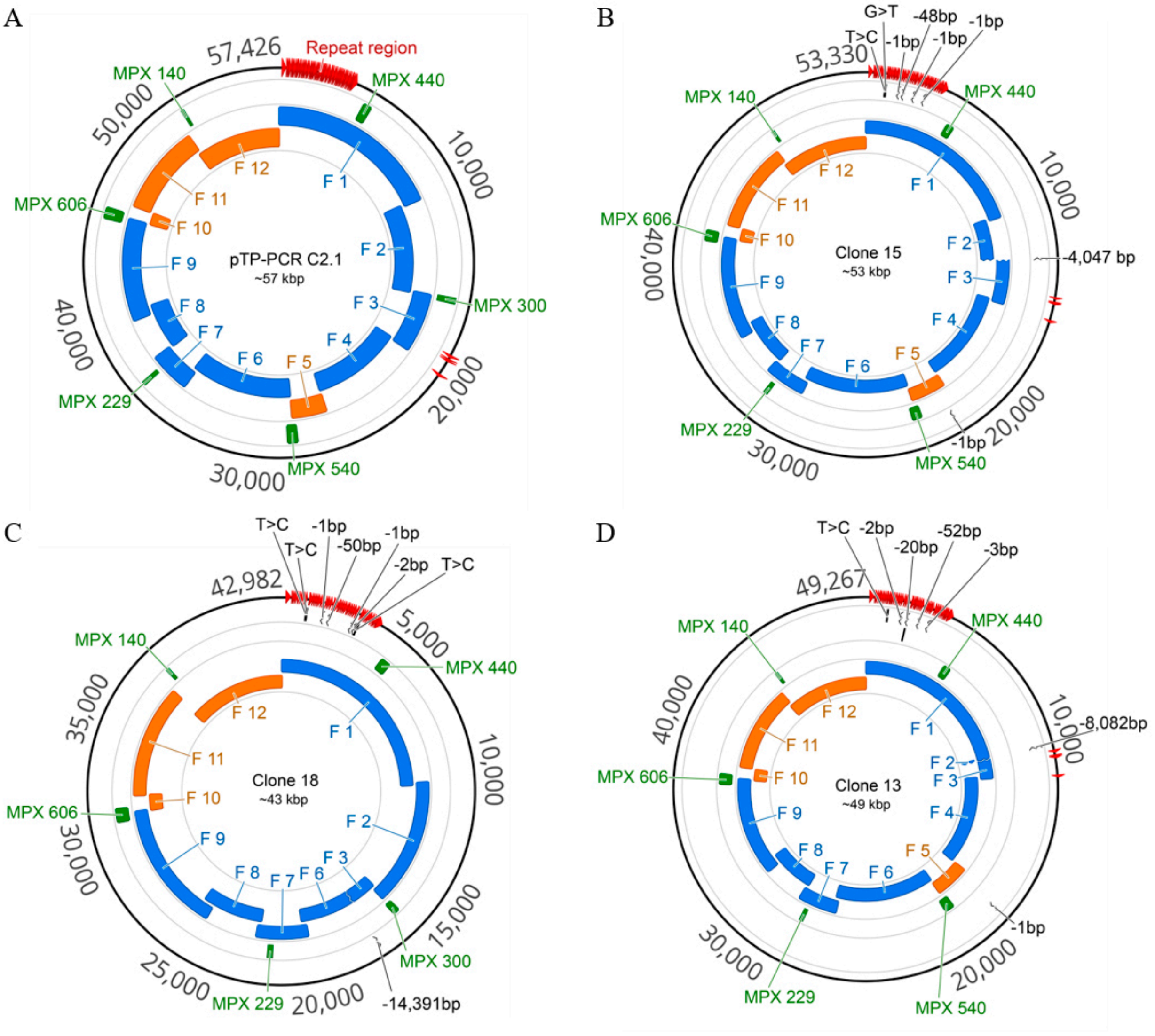

Supplementary Figure S2. Sequencing analysis of mutated pTP-PCR C2.1 clones 13, 15, and 18 identified after propagation in *E. coli*. **A)** Plasmid map of reference sequence, pTP-PCR C2.1, prior to plasmid stability assay (Figure 4) experiment. **B-D)** Plasmid maps of clones 13, 15, and 18 with mutations identified after next generation sequencing displayed. For all plasmid maps the relative sizes and positions of the mitochondrial genome fragments (blue), plasmid backbone fragments (orange), and repetitive sequences (red) are shown. The six multiplex PCR amplicons used for diagnostic screening and their sizes in bp are indicated (green). These images were generated using Geneious version 2020.2.4, created by Biomatters. Notes: 1 – in both **B)** and **D)** a deletion in fragments 2 and 3 resulted in the absent 300 bp multiplex amplicon; 2 – in **C)** a deletion spanning between fragments 3 and 6 resulted in the absent 540 bp multiplex amplicon.

Supplementary Table S1. Primers used in the cloning and screening of all *T. pseudonana* mitochondrial genomes cloned.

Name	Primers	Length (bp)
	**Original Amplification of the Full *T. pseudonana* Mitochondrial Genome**<br>**pTP-PCR C1/2 – Design 1**	
Fragment 1	**P1F –** TTTCATTAGTTGCAGTCACTCCGCTTTGGTTTGGCGCGCCTCGAGCAACAGGGTACAACC<br>**P1R –** AGCGTTAAAAAGTTTAAAAATTAGCGTATATAAATTTATG	**10735**
Fragment 2	**P2F –** TATCTTATACAATTTTTGCTTTCAGGTAACATTTTATTAT<br>**P2R –** AAAAGAATTTGCCTGTATTTTAACTACAAATTATAGGAAA	**6092**
Fragment 3	**P3F–** AATCTAAAACTATCTTACTTAAAAAACTAATTCAAATTAA<br>**P3R–** ATAAAATAAAAAAGTAAGCTGTTCTTAAGCTTGTTTCGGC	**3610**
Fragment 4	**P4F –** CAGGAATGTTCAATTTTGTTTTAAACAAAGTTTACCAACTTAATCCTATTATAGAAGATA<br>**P4R –** GGCCTGCAACGTTCGCTACCTTAGGACCGTTATAGTTACGTAAAGTGTTATAAATTCTAA	**6274**
Fragment 5	**P5F –** TGACCAAGATATAAAATACTTTAGAATTTATAACACTTTACGTAACTATAACGGTCCTAA<br>**P5R –** TTAAATGTTTAAATACTGAGTCGTTTAAAGTTTTGACCATCTATATTACCCTGTTATCCC	**2152**
Fragment 6	**P6F –** TCGTTTCATCGAGTTACGCTAGGGATAACAGGGTAATATAGATGGTCAAAACTTTAAACG<br>**P6R –** TTCCTTTCCAACAACACGAAGCAAATTTTAATAATCATAAGATTATAGTGGATAATTTAG	**7035**
Fragment 7	**P7F –** AAAATTTAAACATTTACCCACTTTCTAGTGATATTCTTGG<br>**P7R –** ATTTTGATGAGCTCTGGTTTAAAATCTTTTAAAACATTGC	**2512**
Fragment 8	**P8F –** TGGTTACGATACTTTGTATAAAAAAAAATTCGAGGGATGGA<br>**P8R –** CGATTTATTGATAAGCAACGAGATTGGTAAATTAGGCCAC	**3250**
Fragment 9	**P9F –** CCATACTCATTTGAATGTTTCGAGTTTATAAATTTGCCAAAAGTACTAAGTTTTAGGTCA<br>**P9R –** CGCTATAATGACCCCGAAGCAGGGTTATGCAGCGGAAGATCTCGAGGCAGTTCAGATTAA	**6216**
Fragment 10	**P10F –** CATAAAAACAGTTCAAATAATTAATCTGAACTGCCTCGAGATCTTCCGCTGCATAACCCT<br>**P10R –**<br>CATAGACGGCCGCCAGCCCAGCGGCGAGGGCAACCAGCTCGCGATCGCGATCGTCTTGCC	**859**
Fragment 11	**P11F –**<br>AGTACATCACCGACGAGCAAGGCAAGACGATCGCGATCGCGAGCTGGTTGCCCTCGCCGC<br>**P11R –** CCATCTGCTCATCATCCAGCTCGCCAACCAGAACACGATAATCACTTTCGGTAAGTGCAG	**5367**
Fragment 12	**P12F –** TGACCAGGAGCTGCTTACTGAGGACGCACTGGATGATCTCATCCCTTCTTTTCTACTGAC<br>**P12R –**<br>AAGTAAGGTTATAAATCATTGGTTGTACCCTGTTGCTCGAGGCGCGCCAAACCAAAGCGG	**5870**
	**Amplification of the *T. pseudonana* Reduced Mitochondrial Genome**<br>**pTP-PCR C3/4 – Design 2**	
Fragment 1	**P2F –** TATCTTATACAATTTTTGCTTTCAGGTAACATTTTATTAT<br>**P2R –** AAAAGAATTTGCCTGTATTTTAACTACAAATTATAGGAAA	**6092**
Fragment 2	**P3F –** AATCTAAAACTATCTTACTTAAAAAACTAATTCAAATTAA<br>**P3R –** ATAAAATAAAAAAGTAAGCTGTTCTTAAGCTTGTTTCGGC	**3610**
Fragment 3	**P4F –** CAGGAATGTTCAATTTTGTTTTAAACAAAGTTTACCAACTTAATCCTATTATAGAAGATA<br>**P13R –** TCGTTTAAAGTTTTGACCATTAAAGTGTTATAAATTCTAA	**6254**
Fragment 4	**P13F –** TAATAATCAATGGATTTTAATTTAAAAACCTATAAACGTT<br>**P6R –** TTCCTTTCCAACAACACGAAGCAAATTTTAATAATCATAAGATTATAGTGGATAATTTAG	**7174**
Fragment 5	**P17F –** ATAAAATTACGAAAAAGTACTACCATACCGTCTAGTCGCT<br>**P8R –** CGATTTATTGATAAGCAACGAGATTGGTAAATTAGGCCAC	**5417**
Fragment 6	**P14F –** CCATACTCATTTGAATGTTTCGAGTTTATAAATTTGCCAA<br>**P14R –**<br>CCTAAAAGACATAGCACGCGACAAGCACGAGCGAGATATCCCAATCAAGCTAGTATCGATTAA<br>GCTTGTATAGCTTGATA	**6372**
Fragment 7	**P15F –**<br>CGAGAATAAATAATATTAGTCTTTGTATTTCTCTTATCAAGCTATACAAGCTTAATCGATACTAG<br>CTTGATTGGGATATC<br>**P15R –**<br>AAGCTTGACCGAGAGCAATCCCGCAGTCTTCAGTGGTGTGATGGTCGTCTATGTGTAAGTCACCA<br>ATGCACTCAACGATT	**9136**
Fragment 8	**P16F –**<br>AATCGTTGAGTGCATTGGTGACTTACACATAGACGACCATCACACCACTGAAGACTGCGGGATT<br>GCTCTCGGTCAAGCTT<br>**P16R –**<br>CCTAAAAAAAAATTTTTGAGAATAAGTAATATTAGTCTTTGATCGTTATTTATCAAAAACGATCG<br>TCTTGCCTTGCTCGT	**8441**

Fragment 9	**P18F –** GAGGCACTTCGAGCTGTAAGTACATCACCGACGAGCAAGGCAAGACGATCGTTTTTGATAAATAACGATCAAAGACTAAT<br>**P1R –** AGCGTTAAAAAGTTTAAAAATTAGCGTATATAAATTTATG	**6810**
	**Final Amplification of the *T. pseudonana* Mitochondrial Genome (pTP-PCR Design 1)**	
Fragment 1	**P25F –** AAAATGCATTGGGAAAAAGGTTAAATTTACCCCAACGAAA<br>**P25R –** GTAGAATATAAGGCTGTGGATTATGCCACAGTTTTTGCT	**5138**
Fragment 2	**P26F –** GTAACCAAGTATGCAGTCCCAATTGCGCAGATTTACCTAC<br>**P26R –** CGTTTTTTTATTAAATCTTGCAATTTACTGCAAAGCAACA	**5244**
Fragment 3	**P27F –** TTTGTAGTGTTGCTCTTATTTTAAACAACTTTGCGGTTTT<br>**P27R –** CGTCACTTCCCAGATCCGCGCTTTCTCTGTCCTTCCTGTG	**5230**
Fragment 4	**P28F –** TAAAAAGGCCGTAATATCCAGCTGAACGGTCTGGTTATAG<br>**P28R –** GCTTAAGTAGACTTGAACTACTGACCTTACGCTTATCAGG	**6884**
Fragment 5	**P29F –** GAGGGCATCTTTTTATATTATAATAATTCCCCAACCTCAA<br>**P29R –** ATGTGACGGGCGGTGTGTACAAAGCCAAGGTACGTATTCA	**5959**
Fragment 6	**P30F –** TCTTAGTTCGGATTGTAAGCTGCAACTCGTTTACATGAAG<br>**P30R –** TTAAAGCTATAGCTTTCAATGCTGAATGATTTGAAGAAGG	**5107**
Fragment 7	**P31F –** AATAGTGAAAATTATGTTTTCGAAAATTCTACAGCAGGT<br>**P31R –** TGTTGTAAACTTATTTGATATTTCTTTGTTTGTTGCATTG	**5948**
Fragment 8	**P32F –** ATTGAATCGTTTTACATGCTCCAGGAATGTTCAATTTTGT<br>**P32R –** ATATTTATTTTATTGTTAAATATTTGGATTTTCCCAGCTA	**5391**
Fragment 9	**P33F –** AATTTAGCTTCGAAATAAAACGCTAAGCCTTGAAAAGATA<br>**P33R –** AGTTGTCTTTTCTGGAAATCGCCATTTGGTCAGTTTTTAG	**5888**
Fragment 10	**P34F –** TACACAAAAGGGAAAGTTAACATTTTCGTAACAATGCAAA<br>**P34R –** CATTTACTCATGAGTTATTTGGGGTAGATTCGTGTGATAA	**6155**
Fragment 11	**P35F –** AGCCATAATCTGTTAAAATACGCTTTAAATTTGAGTGGTT<br>**P35R –** TATGATATGATAGAACATTATAAAACGGTGGAAAGAGGTA	**4289**
	***T. pseudonana* Diagnostic Multiplex PCR Primers**	
Amplicon 1	**P19F –** GCTCACGACATCAGTTTGCT<br>**P19R –** TGCTGGCTTTTCAAGTTCCT	**140**
Amplicon 2	**P20F –** GCTTAATTCACGCTTATTGAAAA<br>**P20R –** ATGGCTTTGAAGGACATCCA	**229**
Amplicon 3	**P21F –** AGTTAAATCTATAGAAAATGCAAATTTAGTTATTAACAGT<br>**P21R –** AAAATAGAATCTTTGAAAAAGTTTCTCCTGAAGTTTATGA	**300**
Amplicon 4	**P22F –** AGTTTTAATTTTTTTTGCTGCTATTGTTTATTTTTTTAGC<br>**P22R –** CACCTAAACTAATGGGTCTAAAAATATATGATATAAGCTC	**440**
Amplicon 5 (only +IR)	**P23F –** TGGCTACCCTGTGGAACACCTACATCTGTATTAACGAAGC<br>**P23R –** GTTTTGGGAACAGGAAGTCATGTGTTCAAATCACATTATT	**540**
Amplicon 6 (only +IR)	**P24F –** GGTCATAAGCCATATATTCAGTAGAATATAAATGTACTAT<br>**P24R –** AGCCGGCCAGCCTCGCAGAGCAGGATTCCCGTTGAGCACC	**606**

Supplementary Table S2. List of mutations identified in cloned *T. pseudonana* mitochondrial genomes by next-generation sequencing.

Position	Mutation	Location/Gene	Effect
		**pTP-PCR C1.1** **Reference: Supplemental File 2**	
888	A > G	Repeat region	Unknown
902	G > A	Repeat region	Unknown
1,000	A > G	Repeat region	Unknown
1,587	28-bp deletion	Repeat region	Unknown
1,879	28-bp deletion	Repeat region	Unknown
1,880	G > C	Repeat region	Unknown
2,112	9-bp deletion	Repeat region	Unknown
2,890	19-bp deletion	Repeat region	Unknown
2,968	T > C	Repeat region	Unknown
3,032	19-bp deletion	Repeat region	Unknown
3,221	A > G	Repeat region	Unknown
3,623	T > C	Repeat region	Unknown
9,841	G > A	*rrnS*	Unknown
21,950	G > T	*rps12*	V87>F
22,337	2-bp deletion	*rps7*	Frameshift
22,433	1-bp deletion	*rps7*	Frameshift
22,716	C > A	*rpl14*	Q44>K
25,274	G > A	*tatC*	T56>I
30,179	1-bp deletion	*rps19*	Frameshift
33,603	T > C	*nad11*	L373S
35,514	G > A	*trnS(gct)*	Unknown
42,462	G > A	*orf718* (*cox1* intron)	No effect
46,617	2-bp deletion	Vector backbone (intergenic)	No effect
56,480	G > A	Vector backbone (intergenic)	No effect
		**pTP-PCR C2.1** **Reference: Supplemental File 2**	
848	A > G	Repeat region	Unknown
862	G > A	Repeat region	Unknown
960	A > G	Repeat region	Unknown
1,549	26-bp deletion	Repeat region	Unknown
1,843	27-bp deletion	Repeat region	Unknown
1,843	G > C	Repeat region	Unknown
2,074	89-bp deletion	Repeat region	Unknown
2,385	31-bp deletion	Repeat region	Unknown
2,743	17-bp deletion	Repeat region	Unknown
2,822	T > C	Repeat region	Unknown
2,886	17-bp deletion	Repeat region	Unknown
3,077	A > G	Repeat region	Unknown
3,479	T > C	Repeat region	Unknown
9,697	G > A	*rrnS*	Unknown
11,838	1-bp deletion	*cox3*	Frameshift
22,763	C > T	*rpl14*	P107>S
27,402	1-bp insertion	Intergenic	No effect
29,153	1-bp deletion	*rpl2*	Frameshift
34,681	1-bp deletion	Intergenic	No effect
41,218	G > A	*orf718* (*cox1* intron)	P388>S
44,615	G > A	*nad5*	No effect
46,475	2-bp deletion	Vector backbone (intergenic)	No effect
49,206	1-bp insertion	Vector backbone (intergenic)	No effect
		**pTP-PCR C3.1** **Reference: Supplemental File 3**	
1,846	1-bp deletion	Vector backbone (*S. meliloti repC2*)	Frameshift
7,209	G > T	Vector backbone (*E. coli repE*)	P103>S
21,050	1-bp insertion	*rrnl*	Unknown
23,484	G > A	*rrnS*	Unknown
24,048	G > A	*nad6*	M142>I

24,115	T > A	*nad6*	L165>M
34,435	C > G	*rps4*	I162>M
35,699	1-bp deletion	*rps12*	Frameshift
39,198	1-bp deletion	Intergenic	No effect
40,103	1-bp deletion	*rps11*	Frameshift
45,855	1-bp insertion	*nad11*	Frameshift
46,604	10,890-bp deletion	Several coding regions	Unknown
**pTP-PCR C4.1**			
**Reference: Supplemental File 3**			
7-8	AC > TA	Vector backbone (intergenic)	No effect
748	C > T	Vector backbone (*NAT*)	P50>L
2,513	C > T	Vector backbone (*S. meliloti repC2*)	R176>H
11,179	G > A	Vector backbone (*E.coli parB*)	No effect
11,228	C > A	Vector backbone (*E. coli parB*)	A146>D
18,322	1-bp deletion	*atp6*	Frameshift
23,483	G > A	*rrnS*	Unknown
25,862	C > T	*cox3*	No effect
33,411	5-bp deletion	Intergenic	No effect
41,950	C > T	*rps3*	S64>L
42,081	1-bp deletion	*rps3*	Frameshift
46,598	G > A	Intergenic	No effect

Supplementary Table S3. Count tables for raw RNA sequencing reads for strains with either the pPT-PCR C2.1 plasmid or vector backbone alone (pPtGE31). Raw counts were enumerated by mapping against the appropriate reference and counted using HTSeq using –nonunique all mode. Three biological replicates were performed (repA, repB, repC) for each condition. Genes regions were counted for the features.

Gene	pPtGE31 repA	pPtGE31 repB	pPtGE31 repC	pPT-PCR C2.1 repA	pPT-PCR C2.1 repB	pPT-PCR C2.1 repC
CAT	2224	4737	3625	3395	4988	6646
atp6	0	0	0	13	6	18
atp8	0	0	0	0	0	0
atp9	0	0	0	0	0	0
cob	0	0	0	51	82	133
cox1	0	0	0	1417	1576	3028
cox2	0	0	0	4	6	23
cox3	0	0	0	12	15	23
nad1	0	0	0	7	9	11
nad11-a	0	0	0	2	6	7
nad11-b	0	0	0	8	8	10
nad2	0	0	0	25	54	51
nad3	0	0	0	1	0	3
nad4	0	0	0	8	10	27
nad4L	0	0	0	0	0	0
nad5	0	0	0	11	17	17
nad6	0	0	0	9	4	17
nad7	0	0	0	29	32	52
nad9-rps14	0	0	0	4	7	11
rRNA 1.1	0	0	0	8	19	22
rRNA 1.2	0	0	0	81	99	165
rRNA 1.3	0	0	0	6	7	12
rRNA 1.4	0	0	0	38	78	145
rpl14	0	0	0	2	2	1
rpl16	0	0	0	0	2	3
rpl2	0	0	0	2	0	3
rpl5	0	0	0	0	0	0
rpl6	0	0	0	0	2	3
rps10	0	0	0	1	0	3
rps11	0	0	0	0	0	4
rps12	0	0	0	0	1	1
rps13	0	0	0	1	0	0
rps19	0	0	0	1	0	4
rps2	0	0	0	0	1	2
rps3	0	0	0	2	2	5
rps4	0	0	0	0	1	7
rps7	0	0	0	1	1	2
rps8	0	0	0	1	0	5
tatC	0	0	0	11	9	38
trnA(ugc)	0	0	0	1	0	0
trnC(gca)	0	0	0	0	1	0
trnD(guc)	0	0	0	0	0	0
trnE(uuc)	0	0	0	19	33	42
trnF(gaa)	0	0	0	1	0	4
trnG(gcc)	0	0	0	0	0	0
trnH(gug)	0	0	0	0	0	0
trnI(cau)	0	0	0	2	0	0
trnI(gau)	0	0	0	2	5	6
trnK(uuu)	0	0	0	0	0	0
trnL(uaa)	0	0	0	0	0	0
trnL(uag)	0	0	0	0	0	1
trnM(cau)	0	0	0	16	26	48
trnM(cau)	0	0	0	1	2	14

trnN(guu)	0	0	0	1	0	1
trnP(ugg)	0	0	0	0	0	0
trnQ(uug)	0	0	0	9	5	14
trnR(ucg)	0	0	0	0	0	1
trnR(ucu)	0	0	0	0	1	5
trnS(gct)	0	0	0	6	15	32
trnS(tga)	0	0	0	0	0	0
trnV(uac)	0	0	0	54	81	121
trnW(cca)	0	0	0	0	0	0
trnY(gua)	0	0	0	0	0	2

Supplementary Table S4. Count tables for raw RNA sequencing reads for strains with either the pTP-PCR C2.1 plasmid or vector backbone alone (pPtGE31). Raw counts were enumerated by mapping against the appropriate reference and counted using HTSeq using –nonunique all mode. Three biological replicates were performed (repA, repB, repC) for each condition. Genes regions were counted for the features.

Genes	pPtGE31 repA	pPtGE31 repB	pPtGE31 repC	pTP-PCR C2.1 repA	pTP-PCR C2.1 repB	pTP-PCR C2.1 repC
CAT	2224	4737	3625	1856	1439	4337
atp6	0	0	0	0	0	1
atp8	0	0	0	0	0	0
atp9	0	0	0	0	0	1
cob	0	0	0	10	3	13
cox1	0	0	0	427	463	914
orf718	0	0	0	402	438	883
cox2	0	0	0	2	0	3
cox3	0	0	0	11	12	27
nad1	0	0	0	2	0	2
nad11	0	0	0	2	0	2
nad2	0	0	0	0	1	1
nad3	0	0	0	1	0	1
nad4	0	0	0	1	0	3
nad4L	0	0	0	0	0	0
nad5	0	0	0	5	2	5
nad6	0	0	0	1	0	6
nad7	0	0	0	46	27	38
nad9	0	0	0	0	0	0
rpl14	0	0	0	0	0	6
rpl16	0	0	0	0	5	1
rpl2	0	0	0	1	12	8
rpl5	0	0	0	0	0	1
rpl6	0	0	0	0	0	0
rps10	0	0	0	0	0	0
rps11	0	0	0	0	0	0
rps12	0	0	0	4	0	0
rps13	0	0	0	1	0	1
rps14	0	0	0	0	0	0
rps19	0	0	0	1	9	3
rps2	0	0	0	0	0	0
rps3	0	0	0	1	0	0
rps4	0	0	0	0	0	0
rps7	0	0	0	0	0	1
rps8	0	0	0	0	0	0
rrnS	0	0	0	23	17	36
rrnl	0	0	0	70	46	112
tatC	0	0	0	2	0	1
trnA(ugc)	0	0	0	1	0	0
trnC(gca)	0	0	0	0	0	0
trnD(guc)	0	0	0	0	0	0
trnE(uuc)	0	0	0	0	0	2
trnF(gaa)	0	0	0	0	0	0
trnG(gcc)	0	0	0	0	0	1
trnH(gug)	0	0	0	0	0	0
trnI(cau)	0	0	0	2	0	0
trnI(gau)	0	0	0	0	0	0
trnK(uuu)	0	0	0	0	0	1
trnL(uaa)	0	0	0	0	0	0
trnL(uag)	0	0	0	1	0	0
trnM(cau)	0	0	0	7	4	15
trnN(guu)	0	0	0	1	0	0
trnP(ugg)	0	0	0	0	0	0

trnQ(uug)	0	0	0	0	0	1
trnR(ucg)	0	0	0	0	0	1
trnR(ucu)	0	0	0	0	0	0
trnS(gct)	0	0	0	1	0	2
trnS(tga)	0	0	0	1	0	0
trnV(uac)	0	0	0	0	0	1
trnW(cca)	0	0	0	1	0	1
trnW(uca)	0	0	0	0	0	1
trnY(gua)	0	0	0	0	0	0

Supplementary Note S1. Determination of outliers in the calculation of doubling time for *E. coli* strains. The median of the dataset was calculated, as well as the lower (Q1) and upper (Q3) quartiles representing that data points at which 25% of the data falls below and above, respectively. The interquartile range (IQR = Q3 - Q1) that indicates the boundaries of non-outlier data points was then calculated. Next, the inner fence of the dataset was found by multiplying IQR by 1.5, then subtracting that value from Q1 and adding it to Q3. Any point outside the inner fence is considered a minor outlier. The outer fence of the dataset was found by multiplying IQR by 3, then subtracting that value from Q1 and adding it to Q3. Any data point outside the outer fence is considered a major outlier. Here, we have only omitted major outliers from our determination of doubling time.
